# A baseline audit of post-vasectomy follow-up at three Cape Town district health facilities

**DOI:** 10.4102/safp.v66i1.6003

**Published:** 2024-10-04

**Authors:** Michael L. le Roux, Renaldo Christoffels, Roland Kroukamp, Jennie Morgan, Omotayo S. Alaofin, Tasleem Ras, Klaus B. von Pressentin

**Affiliations:** 1 Division of Family Medicine, Department of Family, Community and Emergency Care, Faculty of Health Sciences, University of Cape Town, Cape Town, South Africa; 2 Metro Health Services, Western Cape Department of Health and Wellness, Cape Town, South Africa

**Keywords:** vasectomy, clinical audit, quality improvement, access to healthcare, health services delivery, clinical governance, global surgery, primary care urology

## Abstract

**Background:**

Our study focuses on vasectomies, an underutilised contraception method worldwide. Little is known about post-vasectomy semen analysis (PVSA) adherence in our setting, which is an essential step in confirming the procedure’s success. We aimed to describe patient adherence to post-vasectomy follow-up and the success of procedures performed by different surgeon categories at three Cape Town district health facilities.

**Methods:**

We conducted a retrospective descriptive audit. We extracted sociodemographic and procedural information from theatre records and patient folders. The PVSA results were retrieved from Groote Schuur Hospital’s Reproductive Medicine Unit.

**Results:**

The records of 270 patients who underwent vasectomies in local district-level facilities from September 2016 to July 2021 were included. Only 122 (45.2%) semen analysis results were retrievable, of which 115 (94.2%) showed that the procedure was successful. Incomplete patient records significantly impacted the study. A data-collection instrument and implementing standardised stationery were developed, which some sites already use. These measures are designed to ensure more comprehensive datasets for future audits.

**Conclusion:**

The study’s findings have identified flaws in record-keeping practices at the three study sites, a crucial step towards improving post-vasectomy care. Tracking procedural success and patient adherence to post-vasectomy semen analyses using the implemented stationery may assist future research and help drive quality improvement projects.

**Contribution:**

This audit strengthens our understanding of improving this underutilised family planning option in the district health services. In partnership with the local teams, a revised clinical care pathway was developed to inform the delivery of an evidence-informed vasectomy service.

## Introduction

The World Health Organization (WHO) states that access to high-quality and affordable sexual and reproductive health services is fundamental for all.^1^ Different contraceptive methods exist and are classified as short-acting (hormonal and barrier methods), permanent or non-reversible, long-acting reversible contraception (LARC) and traditional methods (such as withdrawal and rhythm methods).^2^ The non-reversible options include male (vasectomy) and female (bilateral tubal ligation) sterilisation.^2^ According to survey-based estimates published by the United Nations (UN) in 2022, the most commonly used contraceptive method by women in the reproductive age range (15–49 years) is female sterilisation (22.9%), followed by male condoms (21.8%).^2^ Vasectomies are generally considered an underutilised modern method of contraception in the world. Despite the safety, effectiveness and permanence of vasectomy, the use of this method has plateaued globally.^3^ Vasectomies are underutilised in low- and middle-income countries, and South Africa and Rwanda were the only African countries with a vasectomy prevalence above 0.1% in a recent analysis of 84 low- and middle-income countries, which concluded that vasectomy use is 61% lower than two decades ago in these countries.^4^

Three techniques have been described, including conventional vasectomy (a scalpel is used to make a 1.5 cm – 3 cm long midline incision), the no-scalpel technique and the minimally invasive technique. The no-scalpel technique is generally used in South Africa, per the European Association of Urology guidelines.^5^ The no-scalpel technique causes less procedure-related discomfort, fewer post-operative complications such as haematoma infection and less post-operative pain.^6^ In terms of the care pathway, accessing a vasectomy starts with extensive pre-procedural counselling. Patients receive an appointment for the procedure and are counselled again as part of the informed consent process. The counselling includes information on the procedure, the risk of complications, the success rate and the need for a post-vasectomy follow-up plan, including the importance of post-vasectomy semen analysis (PVSA) in confirming the procedure’s success. The preferred timing of the PVSA following the procedure is 3 months, in which at least 20 ejaculations should have occurred. The American Urological Association provided guidelines for the interpretation of the PVSA.^6^ The procedure’s success is measured by the absence of sperm in the ejaculate, referred to as azoospermia or the presence of less than 100 000 non-motile spermatozoa per millilitre after 3 months. In the events where the above criteria are not met, it is recommended that semen analysis be repeated at intervals of 6 weeks. Failure of the vasectomy is the presence of motile spermatozoa after 6 months, and it is thus advised that the vasectomy should be redone. International studies identified possible reasons for the non-adherence to PVSA as patients feeling too busy to follow-up, feeling confident that the procedure was correctly conducted and describing the PVSA procedure as too inconvenient.^7^

In the African context, the most recent study (2009) reviewed vasectomies performed between January 2004 and December 2005 at a secondary-level hospital in Cape Town, South Africa.^8^ That study found that vasectomies can be performed safely by junior doctors (in this instance, urology registrars) and that the procedure should be promoted as an effective form of contraception for South African men. More up-to-date information on the service uptake and the PVSA adherence pattern is lacking in our setting. Based on our anecdotal clinical experience, patient adherence to the post-procedure follow-up plan was generally poor. This study can be considered a baseline audit to determine the number and outcome of vasectomies performed at three district health facilities from 2016 to 2021. We sought to assess adherence to the follow-up plan, including PVSA. We were also interested in the association between procedural success and surgeon cadre.

## Research methods and design

### Study design

We conducted a retrospective descriptive audit, analysing the data captured via patient record reviews at three district health facilities offering a vasectomy service in the western half of the Cape Town Metropole, Western Cape.

### Study setting

**FIGURE 1:**
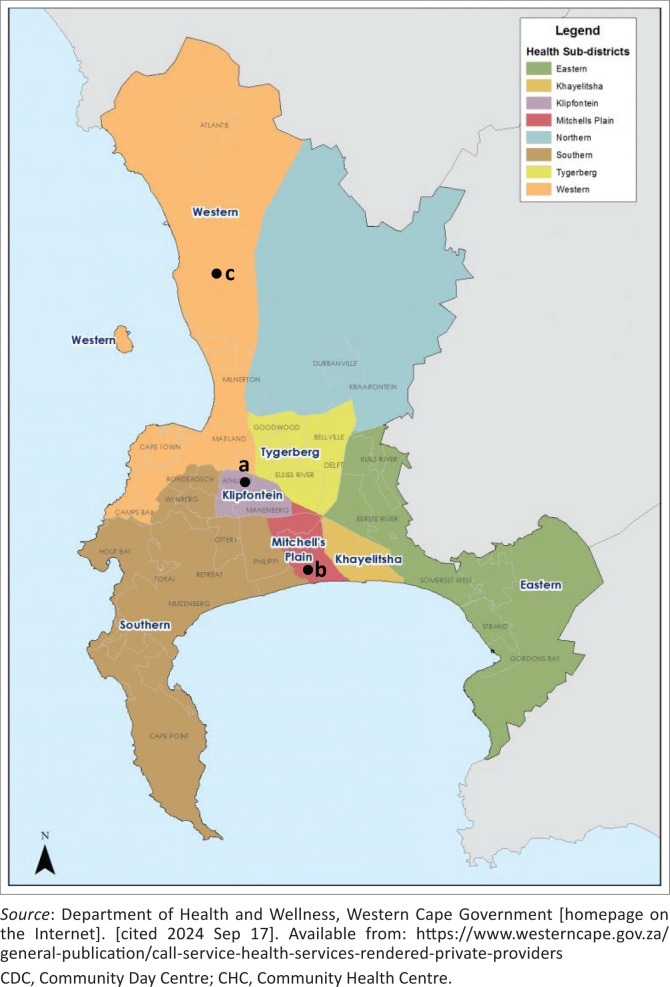
Map of Cape Town Metropolitan Municipality, depicting the location of the three district health services facilities. (a) Heideveld CDC, (b) Mitchell’s Plain CHC, (c) Wesfleur Hospital.

The three facilities included were Wesfleur District Hospital, Mitchell’s Plain Community Health Centre (CHC) and Heideveld CHC. These facilities have a day theatre for minor surgical procedures, where vasectomies are performed by different cadres of skilled doctors, including medical officers, registrars and family physicians. University of Cape Town (UCT)-affiliated family medicine registrars and family physicians perform the procedures at these facilities as part of their outreach service. The location of the facilities is depicted in Figure 1.^9^ The 2011 Census description of the local communities is provided in the next section, as the accessible 2022 Census findings describe the data only at the level of the overall Cape Town metropolitan municipal population, which showed an overall population growth of 1 032 815 (27.6%) from 2011 (3 740 031) to 2022 (4 772 846).^10^

#### Wesfleur Hospital

Wesfleur Hospital (WFH) is a district hospital in Atlantis, and its referral hospitals are in Cape Town, approximately 50 km from Atlantis. The 2011 Census indicates that the population was estimated to be 67 491, mainly Afrikaans-speaking.^11^ The population comprised 68.5% of adults in the working age group (15–64 years old) with a male-to-female ratio of close to 1:1. Households consist of approximately four people on average and only 3.1% of the population older than 20 completed higher education. The average income ranged from R9600.00 to R153 800.00 per annum, and 12.6% had no income. The district hospital is accessible by the community through public transport. Municipal or other transport services are available for travel to the referral hospitals.

#### Mitchell’s Plain Community Health Centre

Mitchell’s Plain also falls under the City of Cape Town Metropole. According to the 2011 Census, the population was 310 485, with an average of approximately four people per household and a male-to-female ratio of almost 1:1.^12^ It was found that 35% of the population over 20 completed matric or higher, and 76% of the labour force (15–64 years old) was employed. The community is served by Mitchell’s Plain CHC, which is accessible through public transport. The CHC’s minor surgical procedures, including the vasectomy service, were suspended in September 2019 because of a fire that destroyed the theatre. The theatre’s renovation has since been completed, and these minor surgical procedures were offered again towards the end of 2022. The CHC refers patients to Mitchell’s Plain District Hospital (MPH), less than 5 km away.

#### Heideveld Community Day Centre

Heideveld is a suburb that falls under the Klipfontein district in the Cape Town metropole. The population was 21 288 in the 2011 Census, with approximately five people per household.^13^ The male-to-female ratio is slightly skewed towards more females. The census found that 31% of those aged 20 years and older completed matric or higher and that 70% of the labour force (15–64 years old) was employed. Heideveld Community Day Centre (CDC) is approximately 10 km from the centre of Cape Town and the referring hospitals, MPH and Groote Schuur Hospital (GSH).

### Study population and sampling strategy

The study population included all the men who underwent vasectomies at these facilities from 2016 to 2021. This timeframe was used as two of the three study facilities introduced the vasectomy service in 2016. We used 2021 as our cut-off time to collect as much data as possible. We included all the available records in this timeframe and no sampling was performed. Like other local public sector hospitals, all three facilities were affected by the coronavirus disease 2019 (COVID-19) pandemic and had to suspend surgical services early in March 2020.^14^ The vasectomy service recommenced around March 2021 when lockdown restrictions were eased.

### Data collection

The first author (M.L.l.R.) collected the data at the three facilities. Given the different modes and practices of record-keeping, the three facilities had different data sources for patient information:

- Wesfleur Hospital used theatre record books to record procedural details. Some electronic copies of the records were incomplete. The semen analyses were not carried out at GSH. Patients were given the option of having them performed privately at their own cost. These results were not recorded in the patient folders.
- Heideveld CDC also used theatre record books. Patient folder review confirmed the existing practice of using standardised stationery related to the procedure, which detailed the pre-procedure counselling, signed consent forms, surgical notes and a follow-up plan. However, no copies of the semen analysis results were available in these patient records.
- Mitchell’s Plain CHC also used theatre record books; however, these books were destroyed in a fire that burned down the theatre in September 2019. No backup system for these records was in place at the facility. With the help of the Reproductive Medicine Unit (RMU) at GSH, we retrieved electronic copies of the results of patients who had vasectomies. We obtained the patients’ details from these electronic records for a folder review.

The information-gathering process consisted of reviewing patient records and, in the case of Mitchell’s Plain CHC, theatre notes kept by the theatre staff. We developed and piloted a data-collection tool with demographic and procedural variables. The demographic data we gathered at the three facilities were retrieved from electronic databases and patient folders. These demographic variables included age, marital status, employment status and the number of children of these patients. Procedural data variables were informed by the guidelines described in the literature review.^6^ The information from the patient folders was captured in a Microsoft Excel spreadsheet.^15^

### Data analysis

This quantitative data were analysed using the Statistical Package for the Social Sciences (SPSS).^16^ The percentages of the different variables and the association between the success of the procedure and the performing surgeon were calculated.

### Ethical considerations

Ethics approval was obtained by the University of Cape Town’s Human Research Ethics Committee (Ref 196/2022). The Western Cape Provincial Health Research Committee (WC_202204_012) approved facility access.

## Results

The study population consisted of 270 patients who underwent vasectomies at the three facilities from September 2016 until July 2021. Table 1 shows the frequency and percentages of the number of procedures performed at the different facilities. Wesfleur Hospital and Mitchell’s Plain CHCs started performing the procedures in 2016. Heideveld CDC has recorded vasectomies since 2018.

**TABLE 1:** Number of vasectomies conducted at the three facilities (*N* = 270).

Facilities	Frequency (*n*)	%
HCDC	82	30.4
MPCHC	89	33.0
WFH	99	36.7

HCDC, Heideveld Community Day Centre; MPCHC, Mitchells Plain Community Health Centre; WFH, Wesfleur Hospital.

### Sociodemographic data of patients

Table 2 presents the sociodemographic data. Most patients were between 30 years and 50 years (78.5%). The age of the patients was unknown in 10.7% of the records. Around half of the patients (53%) were married, and in many patients, the marital status was unknown (45.2%). Thirty-six per cent of the population had three to four children. There was a substantial number of folders in which the number of children was not recorded (36.7%). The folder review also found that all the patients whose employment status was recorded (62.2%) were employed.

**TABLE 2:** Sociodemographic data of the study population included in the audit (*N* = 270).

Variables	Categories	Frequency (*n*)	%
Age (years)	0–29	12	4.4
	30–39	129	47.8
	40–49	83	30.7
	50–59	16	5.9
	60–99	1	0.4
	Unknown	29	10.7
Employment status	Employed	168	62.2
	Unemployed	0	0.0
	Unknown	102	37.8
Marital status	Married	143	53.0
	Divorced	3	1.1
	Widow	1	0.4
	Single	1	0.4
	Unknown	122	45.2
Number of children	≤ 2	45	16.7
	3–4	98	36.3
	> 4	28	10.4
	Unknown	99	36.7

### The number of patients who adhered to follow-up and the success or failure of the procedure as determined by the post-vasectomy semen analysis

Table 3 represents the results of the PVSA performed. The results were obtained from the RMU at GSH, where the semen analyses were performed. The results for two facilities were collected: Heideveld Community Day Centre (HCDC) and Mitchells Plain Community Health Centre (MPCHC).

**TABLE 3:** Post-vasectomy semen analysis results.

Facility	Results of PVSA						Total number
	Successful		Unsuccessful		Unknown		
	*n*	%	*n*	%	*n*	%	
HCDC	27	81.8	6	18.2	49	59.8	82
MPCHC	88	98.9	1	1.1	0	0.0	89
WFH	0	0.0	0	0.0	99	100	99

**Total**	**115**	**94.2**	**7**	**5.8**	**148**	**54.8**	**270**

PVSA, post-vasectomy semen analysis; HCDC, Heideveld Community Day Centre; MPCHC, Mitchells Plain Community Health Centre; WFH, Wesfleur Hospital.

The HCDC theatre records showed 82 vasectomies that were conducted; however, the folder review revealed that only 49 (59.7%) PVSA results were known. This was found to be because of factors such as patient folders that were not retrieved or patients who did not go for their PVSA. The PVSA results revealed that 27 out of 33 known results were successful (81.8%), and six were unsuccessful (18.2%).

The MPCHC facility’s PVSA results were retrieved from the RMU at GSH. An electronic database at the RMU contained 89 PVSA results. These showed that 88 procedures were successful (98.9%) and only one was unsuccessful (1.1%).

The WFH results were unavailable as it was not standard protocol to refer patients for PVSA at the RMU. Ninety-nine vasectomies were recorded at WFH, and upon the folder review, none of the PVSA results were captured in the clinical records.

Thus, only 122 patients (45.1%) had documented adherence to the post-procedure follow-up plan. Of these 122 patients whose PVSA results were known, 115 procedures were successful (94.2%).

### The association between performing surgeons and the success of vasectomy

Table 4 demonstrates that of the 270 vasectomies, family physicians performed almost half (*n* = 123, 45.6%), urologists performed a third (*n* = 98, 36.3%), whereas family medicine registrars (*n* = 24, 8.9%) and medical officers (*n* = 5, 1.9%) contributed a lower number. The missing data from the source documents severely impacted our ability to describe the success percentages obtained by the different surgeon categories, as the surgeon category was unknown in 20 (7.4%) procedures. According to the limited accessible results, urologists had a higher success rate of 97.2% (*n* = 72 out of 74 known results). Family physicians had a lower success rate of 85.7% (*n* = 24 out of 28 known results); however, this success rate was impacted considerably by the high percentage, 77.2% (*n* = 95 out of 123), of unknown data for this surgeon category. The missing data from the patient records review made it very difficult for us to accurately determine the association between the success of the procedure and the performing surgeon.

**TABLE 4:** Surgeon cadre association with post-vasectomy semen analysis outcome.

Surgeon category	Post-vasectomy semen analysis outcome							
	Successful		Unsuccessful		Unknown		Total	
	*n*	%	*n*	%	*n*	%	*n*	%
Family physician	24	85.7	4	14.3	95	64.2	123	45.6
Urologist	72	97.2	2	2.8	24	16.2	98	36.3
Family medicine registrar	2	100	0	0	22	14.9	24	8.9
Medical officer	0	0	0	0	5	3.4	5	1.9
Unknown cadre	17	94.4	1	5.6	2	1.4	20	7.4

**Total**	**115**	**94.2**	**7**	**5.8**	**148**	**54.8**	**270**	**-**

## Discussion

### Key findings

The study revealed that this contraceptive method is available at all three facilities included in the audit. However, our description of procedural success was limited by the incompleteness of the source documents included in this baseline audit. Where data were available, we could show fair procedural success rates. A considerable number of patients did not follow-up for PVSA. Health service strengthening activities have been implemented to address these issues identified in the baseline audit.

### Discussion of the key findings

This audit showed that vasectomies are utilised in our local setting, which is refreshing given that it is an underutilised method.^4^ This uptake may be ascribed to several factors, such as ineffective information sharing at the community level, uncertainty about the procedure’s effectiveness, operation and potential complications and a lack of knowledge on accessing and navigating the care pathway. Our study did not collect data to confirm this hypothesis. All three facilities have health promoters who are responsible for the distribution of information about the procedure. They offer counselling for males who show an interest in undergoing vasectomies.

The global success of vasectomies is currently estimated to be 99%.^17^ Based on the available source data, our findings report that the success at these facilities is below this target (94.2%). The need for a repeat vasectomy globally is less than 1%,^17^ which currently leaves the results from our audit below the target. These results could be because of surgical technique, surgeon experience and unconfirmed vas deferens occlusion (no available histology to confirm the occlusion). The current international and local guidelines do not require histological evidence of vas deferens occlusion.^6^ However, given the available local information, our protocols were updated to include sending specimens (left and right vas deferens) for histological confirmation. This will indicate whether the procedure was successful and whether failure (risk of falling pregnant, which was 1 in 2000) was because of not being 100% reliable.^6^

There have been some international studies that showed that the follow-up after having a vasectomy is generally low, and non-adherence was found to be greater than 30%.^7^ The reasons for the low follow-up percentage have been explored on an international level and include patients feeling too busy to follow-up, patients feeling confident that the procedure was correctly performed and patients describing that the procedure process was too inconvenient.^7^ The reasons for non-adherence have yet to be explored on a local level. Our findings revealed that 45.2% of patients adhered to the recommended follow-up appointment for the PVSA. This puts the non-adherence percentage at 54.8%, keeping with internationally described follow-up rates. However, the percentage of adherence to PVSA may be higher than reported in the WFH group as these patients were encouraged to access a private facility for their PVSA. Another plausible reason for the poor follow-up rate may be that some patients were not well-informed or motivated to adhere to the follow-up protocols. Our facilities are also known to be very busy and full daily. This often leads to long patient waiting times, which may impact adherence. Health promotors at some facilities aim to stay in contact with patients following the procedure via telephone or messaging service and thus ensure they keep to the appointments to have their PVSA performed. This process involves the staff (nurses and health promotors) making appointments at GSH RMU for patients’ follow-up tests. Reports from counsellors at the facilities reveal that the implementation of the appointment system has been beneficial in assuring patient adherence.

The nature of the relationship between the cadre of surgeons who performed the vasectomies and the procedure’s outcome could not be described accurately. It is, however, evident from our findings that the urologists had a relatively larger percentage of success and this could have been because of the implementation and maintenance of the PVSA pathway that was set in place by them and their local teams (theatre staff) in the initial phase of implementing the service. From anecdotal testimony from staff, we found that the theatre nurse’s role in referring patients to GSH RMU ensured that they followed up for their PVSA. At present, family physicians are the only cadre performing vasectomies at these primary care facilities. The available results revealed a suboptimal success rate of 85.7%; however, the unknown PVSA results largely impacted it. These results likely underestimate the success rate as anecdotal reports from facility staff revealed very few failed procedures.

### Implications for ongoing vasectomy service strengthening activities

**FIGURE 2:**
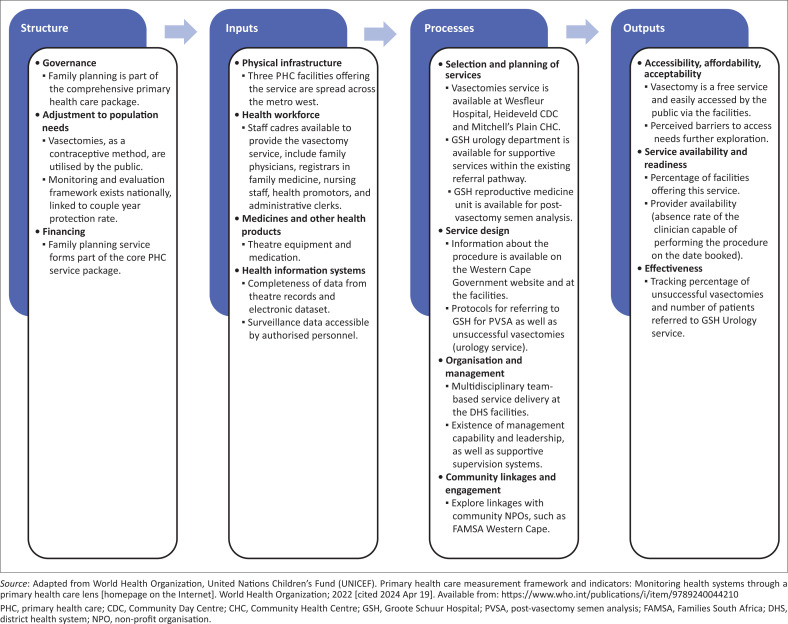
Vasectomy service framework.

The WHO primary health care measurement conceptual framework describes how clinical governance, policies, healthcare workforce and health information systems contribute to quality service delivery and continuity of care.^18^ Figure 2 illustrates a suggested framework based on this WHO framework, which could assist in improving the vasectomy service from a health systems perspective.^18^ The recent call for collaboration between urologists and primary care providers is manifested in the processes domain, given the support of the GSH urology department for care coordination.^19^

A revised clinical care pathway to inform the delivery of an evidence-informed vasectomy service emerged during the audit and was developed in partnership with the local teams (Box 1). Capturing information on an electronic platform can strengthen access to a well-coordinated service through the continuity of clinical records. It would ensure follow-up in cases where patients disengage from the services. Standardised stationery for the vasectomy process is already being used at MPCHC and HCDC. This has since been introduced at WFH to ensure a uniform approach to vasectomies.

BOX 1 Vasectomy clinical care pathway for Cape Town Metro West primary healthcare facilities.

Steps in the proposed vasectomy clinical care pathway

- Patients access information from the Western Cape Government website and from health promotors at the facilities.
- They receive counselling on the procedure and are booked to have the vasectomy conducted.
- Relevant information about the patient is captured in their folders.
- Informed consent is obtained by the surgeon performing the vasectomy.
- The vasectomies are then performed according to guidelines and the post-procedure plan is discussed with the patient.
- Health promotors or theatre staff book appointments at Groote Schuur Hospital Reproductive Medicine Unit for post-vasectomy semen analysis and stay in contact with patients to ensure adherence to the follow-up plan.
- Results are retrieved by authorised personnel and captured in the clinical records.
- Patients are informed of the results and whether the vasectomy was successful or whether it was unsuccessful.
- In the event of an unsuccessful vasectomy, patients are referred to Groote Schuur Hospital Urology Department for further management.

### Study strengths and limitations

Our study represents a baseline audit to illuminate a poorly described family planning service and inform future research. The audit process enabled us to identify gaps in the care pathway process, from recruiting patients for vasectomies to documenting the pre- and post-procedure process. Active engagement with the local role-players facilitated the implementation of enhanced systems and record-keeping, including electronic data-collection tools that we developed and standardised stationery to ensure an efficient process.

Our study is limited by its retrospective cross-sectional observational design, which does not allow for the description of causation between variables. Two major limiting factors in our research were the loss of the MPCHC theatre records because of fire and incomplete record-keeping encountered in the available records. Because of the missing data, we could not accurately describe the possible association between the vasectomies’ success and the surgeons’ cadre.

### Recommendations

Following this audit, record-keeping has now been standardised at all three facilities as part of a clinical care pathway. The adherence to the care pathway should be monitored and evaluated continuously to ensure efficiency. Health promoters should continue to play a significant role in recruiting and counselling patients for vasectomies. They could be allocated as the central person to arrange appointments for PVSA as they represent the ideal nucleus of the vasectomy service, given their role in supporting the patient on their journey, from pre-procedural counselling to post-procedural care. Stronger linkages with community networks and non-profit organisations such as Families South Africa (FAMSA) should be built to assist with counselling for families about the option as a family planning method.

The family physicians at these primary care facilities are currently the main surgical cadre performing the procedure. They are ideally placed to access and review the PVSA results as part of the post-procedural care pathway.

Further research may include an audit of record-keeping, determining the procedural success of the procedure using the PVSA findings, an association between the surgeon and the outcome of the vasectomy and adherence to the follow-up protocol in keeping with international guidelines. Qualitative research is also needed to identify factors that play a role in non-adherence to follow-up and explore perceived barriers to accessing the service.

## Conclusion

Our study shows that vasectomy as a form of contraception is being utilised in our communities. It is evident from this baseline audit that missing records influenced our ability to answer all the study objectives. Patient adherence to the follow-up PVSA was found to be low. Family physicians and family medicine registrars are currently the surgeons who perform vasectomies in primary healthcare settings. The success rate of the procedures performed by these cadres of surgeons could not be established accurately in our study. Information about the quality measures in place to grow this service will strengthen the uptake of the procedure. The data-collection instrument that we implemented in partnership with the local teams will ensure the availability of adequate records to allow for complete datasets in follow-up audits. We hope our experience will encourage other clinicians, managers and researchers to enhance and study access to this vital family planning option in similar settings.
